# Critical Role for Very-Long Chain Sphingolipids in Invariant Natural Killer T Cell Development and Homeostasis

**DOI:** 10.3389/fimmu.2017.01386

**Published:** 2017-11-01

**Authors:** Ashish Saroha, Yael Pewzner-Jung, Natalia S. Ferreira, Piyush Sharma, Youenn Jouan, Samuel L. Kelly, Ester Feldmesser, Alfred H. Merrill, François Trottein, Christophe Paget, Karl S. Lang, Anthony H. Futerman

**Affiliations:** ^1^Department of Biomolecular Sciences, Weizmann Institute of Science, Rehovot, Israel; ^2^Medical Faculty, Institute of Immunology, University Duisburg-Essen, Essen, Germany; ^3^INSERM U1100, Centre d’Etude des Pathologies Respiratoires, Faculté de Médecine, Tours, France; ^4^School of Biology and Petit Institute for Bioengineering and Bioscience, Georgia Institute of Technology, Atlanta, GA, United States; ^5^Life Science Core Facilities, Weizmann Institute of Science, Rehovot, Israel; ^6^Centre d’Infection et d’Immunité de Lille, INSERM U1019, CNRS UMR 8204, University of Lille, CHU Lille- Institut Pasteur de Lille, Lille, France

**Keywords:** very-long chain ceramides, glycosphingolipids, invariant natural killer T cells, ceramide synthase 2, lymphocytic choriomeningitis virus, liver, thymus

## Abstract

The role of sphingolipids (SLs) in the immune system has come under increasing scrutiny recently due to the emerging contributions that these important membrane components play in regulating a variety of immunological processes. The acyl chain length of SLs appears particularly critical in determining SL function. Here, we show a role for very-long acyl chain SLs (VLC-SLs) in invariant natural killer T (*i*NKT) cell maturation in the thymus and homeostasis in the liver. Ceramide synthase 2-null mice, which lack VLC-SLs, were susceptible to a hepatotropic strain of lymphocytic choriomeningitis virus, which is due to a reduction in the number of *i*NKT cells. Bone marrow chimera experiments indicated that hematopoietic-derived VLC-SLs are essential for maturation of *i*NKT cells in the thymus, whereas parenchymal-derived VLC-SLs are crucial for *i*NKT cell survival and maintenance in the liver. Our findings suggest a critical role for VLC-SL in *i*NKT cell physiology.

## Introduction

Natural killer T (NKT) cells are a subset of T lymphocytes that act at the interface between the innate and adaptive immune systems (1). A subset of NKT cells, termed invariant NKT (*i*NKT) cells, is found at low levels (<1% of hematopoietic cells) in many organs but is highly enriched in the liver, where it represents up to 30% of resident T lymphocytes (2). *i*NKT cells express a restricted T cell receptor (TCR) repertoire composed of an invariant TCR α chain (Vα14-Jα18 in mice and Vα24-Jα18 in humans) paired with a limited number of β chains (Vβ8.2, Vβ7, or Vβ2 in mice and Vβ11 in humans). *i*NKT TCRs recognize CD1d-restricted glycolipid ligands, among them a glycosphingolipid (GSL), α-galactosylceramide (α-GalCer), which was originally isolated from a marine sea sponge. Reactivity to α-GalCer is used to define *i*NKT cells and distinguishes them from other NKT subsets (3). Most mammalian GSLs exist as β anomers although there is some evidence that mammalian α-linked glycosylceramides are also found (4, 5).

Glycosphingolipids consist of a ceramide backbone to which various glycans are added at the C1 position. Ceramide itself is comprised of a sphingoid long chain base to which a fatty acid is *N*-acylated, with each of the six mammalian ceramide synthases catalyzing the addition of fatty acids with different acyl chain lengths (6, 7). The acyl chain length appears particularly critical in determining SL function (8). Specifically, very-long acyl chain (VLC) GSLs have been studied extensively in immune cell regulation (9–13), and it was suggested that VLC-GSLs, such as C24:1-glucosylceramide (GlcCer) might serve as self-antigens required for thymic *i*NKT cell maturation (4, 14, 15).

In the current study, we take advantage of a mouse which is unable to generate VLC-sphingolipids (SLs) [with a C22-C24 acyl chain length (16)] to study the impact on *i*NKT cell homeostasis. Ceramide synthase 2 (CerS2)-null mice display liver (17–19), brain (20), lung (21, 22), and adrenal gland pathologies (23). These disorders are caused by a variety of factors including increased cellular turnover of hepatocytes (17), dysfunction of specific membrane proteins and receptors (18, 24–26), impaired mitochondrial complex IV activity (19), demyelination (20), chronic lung inflammation (21), and oxidative stress (23), which can be caused either by changes in levels of specific SLs or by changes in the biophysical properties of membranes (27).

We demonstrate that CerS2-null mice display a significant reduction of *i*NKT cells in the thymus and liver, which render CerS2-null mice susceptible to infection with a hepatotropic strain of the lymphocytic choriomeningitis virus (LCMV-WE); susceptibility to infection is overcome upon adoptive transfer of wild-type (WT) *i*NKT cells. Our data support the notion that VLC-GSLs act as endogenous self-ligands for *i*NKT cell development in the thymus (4). Moreover, akin to other naïve lymphocytes which depend on low affinity self-ligand exposure for survival (28, 29), *i*NKT cells require similar parenchymal survival signals in the liver.

## Materials and Methods

### Reagents and Antibodies

The following antibodies were from Bio-Legend (San Diego, CA, USA): anti-CD16/32 FcγR (clone 93); Pacific blue anti-mouse CD45 (clone 30-F11); allophycocyanin (APC) anti-mouse CD45.1 (clone A20); APC or phycoerythrin (PE)/Dazzle 594 anti-mouse CD3 (clone 17 A2); PercpCy5.5 anti-mouse CD4 (clone GK1.5); fluorescein isothiocyanate anti-mouse CD8a (clone 53-6.7); Alexa Fluor 700 anti-mouse CD44 (clone 1M7); PE-Cy7 anti-mouse CD24 (clone M1/69); Pacific blue anti-mouse CD1d (clone 1B1); APC-Cy7 anti-mouse CD19 (clone 6D5); PE anti-mouse Ly6G (clone 1A8); Pacific blue anti-mouse Ly6C (clone HK1.4); APC-Cy7 anti-mouse CD11b (clone M1/70); PE-Dazzle anti-mouse CD11c (clone N418); PE anti-mouse B220 (clone RA3-6B2); and PercpCy5.5 anti-mouse PDCA1 (clone 927). An APC anti-mouse IFNγ antibody (clone XMG1.2) and APC-streptavidin were from eBioscience, MA, USA. A biotin-anti-mouse-NK1.1 antibody (clone PK136) was from Bio-gems International Inc., CA, USA. PE anti-mouse F4/80 (clone Cl:A3-1) was from AbD Serotec, Oxford, UK, and PE- or Alexa 488-anti-mouse CD1d-PBS57 tetramer (PBS-57 is a synthetic analog of α-GalCer) was provided by the NIH tetramer core facility at Emory University, Atlanta, GA, USA. Rat anti-LCMV serum was made in-house and the Cy3 donkey anti-rat antibody was from Jackson ImmunoResearch Laboratories Inc., PA, USA.

An IFN-α ELISA kit was from Thermo Fischer Scientific Inc., USA, anti-PE magnetic microbeads were from Miltenyi Biotec, Bergisch Gladbach, Germany, an RNeasy mini kit was from Qiagen (Venlo, Netherlands), a qScript™ C-DNA synthesis kit was from Quanta Biosciences Inc. (MA, USA), the Perfecta SYBR Green fastMix was from Quanta Biosciences Inc. (MA, USA), and a IL-2 ELISA kit was from R&D systems (Minneapolis, MN, USA). α-GalCer was produced as described (30). Brefeldin A was from Sigma-Aldrich (St. Louis, MO, USA).

### Mice

Ceramide synthase 2 null and WT littermate control mice were generated by crossing C57BL/6 CerS2^+/−^ mice with 129S2v/Jae CerS2^+/−^ mice (16). Mice were housed in a specific pathogen-free barrier facility at the Weizmann Institute of Science. Experimental procedures were performed in accordance with guidelines approved by the Institutional Animal Care and Use Committee.

### LCMV-WE Virus Infection

LCMV-WE was propagated in baby hamster kidney 21 cells. The viral titer was determined by a plaque assay (31). Two million plaque-forming units (PFU) of LCMV-WE were injected intravenously through the retro-orbital plexus into CerS2-null mice. Mice were sacrificed 2 and 6 days later and their livers and spleens removed. The big lobe of the liver was fixed in optimal cutting temperature freezing medium and the rest of the liver frozen at −80°C. Liver sections (5 µm) were cut using a cryostat (Leica Systems, Nussloch, Germany) and dried overnight at room temperature. Sections were used for anti-LCMV staining or stored at −20°C. Sections were fixed with acetone for 10 min and blocked with 10% fetal bovine serum (FBS) for 1 h. Sections were stained with rat anti-LCMV serum for 1 h followed by three washings with PBS. Sections were incubated with a Cy3-conjugated donkey anti-rat antibody (1:700 in 1% FBS) and counterstained with Hoechst 33342 (Molecular Probes, OR, USA) for 5 min and washed three times with PBS. Sections were mounted using Fluoromount G mounting medium (Southern Biotech, AL, USA). Slides were dried overnight and images were captured using a Nikon eclipse Ti-s inverted microscope. Images were processed using NIS elements 3.0 imaging software (Nikon Instruments Inc., NY, USA).

### *In Vitro* Restimulation of LCMV-Specific T Cells

For intracellular cytokine staining, isolated liver cells were incubated with or without the LCMV-specific peptides, GP33 (0.1 µM), or GP64 (1 µM). After 1 h, brefeldin A (5 µg/ml) was added to block cytokine secretion (32), followed by an additional 5 h incubation at 37°C. After surface staining with anti-CD8 or anti-CD4 antibodies, cells were fixed with 2% formalin and permeabilized with PBS containing 1% fetal calf serum (FCS) and 0.1% saponin, and stained with an anti-IFN-γ antibody for 30 min at 4°C (32).

### RNA-seq Processing and Analysis

Liver was homogenized in 750 µl Trizol reagent (Invitrogen) and vortexed after adding 150 µl chloroform and incubated for 5 min at room temperature, followed by centrifugation for 15 min at 20,000 *g*. 300 µl from the upper phase was transferred to a new microtube and after the addition of 70% ethanol (1:1 vol), transferred to an RNeasy column (RNeasy mini kit, Qiagen). RNA was purified following DNase I digestion on the column according to manufacturer’s instructions. For library preparation, 6.1 µl of total RNA (5 ng/µl) was used for the MARS-seq protocol (33). RNA-seq libraries were sequenced using Illumina NextSeq-500. For analysis, adapters and low quality bases were removed from the raw reads using cutadapt (34). The remaining reads were mapped to the *Mus musculus* genome (mm10) using STAR v2.4.2a (35) with the option alignEndsType EndToEnd. Only reads with unique mapping were considered for further analysis. Gene expression levels were calculated using htseq-count (36) with option intersection-strict and mm10 Refseq 3′UTR GTF annotations. Duplicate reads were filtered if they mapped to the same gene and had identical UMIs. Normalization and differential expression analysis was performed using the DESeq2 R-package (Bioconductor, https://bioconductor.org/packages/release/bioc/html/DESeq2.html). Differentially expressed genes were defined as genes that had a significant adjusted *p* value (<0.05) and at least twofold change. Differentially expressed genes in at least one of the comparisons were clustered using the *k*-means algorithm (Partek^®^ Genomics Suite software, Partek Inc., St. Louis, MO, USA) with Pearson’s dissimilarity as the distance metric. The value of *k* was evaluated by the Davies–Bouldin criterion for a range of possible values (1–20) and visual inspection of local minimums. Heatmaps were drawn with Partek.

### Quantitative Real-time PCR

Total RNA was isolated using an RNeasy mini kit according to manufacturer’s instructions. cDNA synthesis was performed using a QScript™ C-DNA synthesis kit and qPCR performed using the Perfecta SYBR Green fastMix and an ABI Prism 7000 Sequence Detection System (Applied Biosystems, Life Technologies). The sequence of real-time primers for LCMV-glycoprotein was, forward, 5′CGCACCGGGGATCCTAGGC 3′, reverse, 5′ATACTCATGAGTGTATGGTC 3′. The following primers were purchased from Qiagen Inc., with catalog numbers indicated: GAPDH, QT01658692; MX1, QT01064231; IRF7, QT00245266; OAS1, QT01056048; ISG15, cat QT02274335; Bst2, QT01066184; and Usp18, QT00167671. The sequence of primers used for the validation of differentially expressed genes found in RNAseq analysis was: *Orm2*, forward, 5′TGGAAGCTCAGAACCCAGAAC 3′, reverse 5′ GCCGGTAATCAGGGTTTAGG 3′; *Saa2*, forward, 5′ CTAGGAACACTGAAGATGCTCTC 3′, reverse 5′TCTCCTCCTCAAGCAGTTACTA 3′; *Saa3*, forward 5′AGCCAAAGATGGGTCCAGTT 3′, reverse, 5′ TAGGCTCGCCACATGTCTCT 3′; *Hpx*, forward, 5′ GGGAGAGTTGCCGAAGTTGA, reverse, 5′CCTCCACACAAACTCCCCTTT 3′; *Hp*, forward, 5′ TTCTACAGACTACGGGCCGA 3′, reverse, 5′ CCCACACACTGCCTCACATT 3′; *Apcs*, forward, 5′GCTACGTAGTCATCAGGCCC 3′, reverse, 5′GACCTCTTACACATCGGCCA 3′.

### Flow Cytometry

Approximately 10^6^ cells were blocked with an anti-CD16/32 FcγR antibody for 10 min and subsequently stained with fluorescently labeled antibodies for 10 min. Flow cytometry was performed on an LSR II flow cytometer (BD Biosciences, San Jose, CA, USA) and analyzed using Flow Jo V10 software (Flow Jo, LLC, Ashland, OR, USA).

### Hepatocytes and Mononuclear Cell (MNC) Isolation

Hepatocytes were isolated as described (37). For isolation of MNCs, the supernatant obtained after separating hepatocytes was centrifuged at 300 *g* to pellet MNCs. Erythrocytes were lysed with ammonium chloride, potassium (ACK) buffer (150 mM NH_4_Cl, 10 mM KHCO_3_, 0.1 mM EDTA, pH 7.2), and dead cells separated on a 40% Percoll gradient by centrifugation (30 min, 300 *g*). The pellet containing MNCs was resuspended in PBS containing 1% BSA and cells counted using a hemocytometer.

Splenocytes and thymocytes were isolated after mechanical disruption in PBS containing 1% BSA followed by filtration through a 70-µm cell strainer. Splenocytes were incubated in ACK buffer for 2 min to lyse erythrocytes. Live cells were counted in a hemocytometer using trypan blue. Blood was withdrawn retro-orbitally using capillaries containing anti-coagulating agents. Erythrocytes were lysed using ACK lysis buffer for 2 min. The cells were washed, suspended in PBS, and counted in a hemocytometer.

### Generation of Bone Marrow (BM)-Derived Cells and *In Vitro i*NKT Cell Activation Assay

Bone marrow-derived cells were cultured in Iscove’s Modified Dulbecco’s Medium supplemented with 10% FCS with 1% (of total volume) of a supernatant from a granulocyte-macrophage colony-stimulating factor (GM-CSF)-expressing cell line (J558-GM-CSF) (38). Dendritic cells (DCs) were used on day 14 of culture. This protocol yielded >90% cell purity as evaluated by FACS (DCs were identified by cell surface expression of CD11c^+^ MHC II^+^). DCs (1 × 10^5^ cells/ml) were cocultured with 1 × 10^5^ cells of a mouse type I NKT cell hybridoma (DN32.D3) in the presence of vehicle or various concentrations of α-GalCer (10–100 ng/ml) in complete Roswell Park Memorial Institute media supplemented with 5% FCS for 24 h. Supernatants were collected and the IL-2 concentration measured by ELISA (R&D systems, MN, USA).

### *i*NKT Cell Enrichment and Adoptive Transfer

Invariant NKT cells from liver MNCs were enriched using anti-PE microbeads (Miltenyi Biotec, Bergisch Gladbach, Germany). Briefly, MNCs from livers obtained from at least seven mice were isolated, pooled, and blocked with an anti-CD16/32 antibody for 10 min at 4°C in PBS containing 1% BSA. Ten microliters each of a PE-conjugated CD1d-PBS57 tetramer were added per 10^7^ cells in 100 µl of magnetic-activated cell sorting (MACS) buffer (PBS containing 0.5% BSA and 2 mM EDTA) and incubated for 10 min in the dark at 4°C. The cells were washed and resuspended in 80 µl of PBS to which 20 µl of anti-PE microbeads per 10^7^ cells were added and kept in the dark for 15 min at 4°C. Cells were washed and resuspended in 500 µl of MACS buffer. Cells were passed through a magnetic separation column followed by three washings to remove unbound cells. The magnetic labeled-bound cells were released from the column by removing the magnet and flushing the column with a syringe plunger. The purity of enriched CD3^+^ and CD1d/PBS-57 tetramer^+^ cells was determined by flow cytometry. One million *i*NKT cells were washed and resuspended in 200 µl of PBS. Cells were injected i.v. through the retro-orbital plexus into CerS2-null mice. As a control, an equal amount of PBS was injected into CerS2-null mice.

### Generation of BM Chimeras

C57BL/6 CD45.1^+^ WT mice were crossed with WT 129S4/SvJae CD45.2^+^ mice to generate WT C57BL/6;129S4/SvJae F1 mice, which carry both CD45.1 and CD45.2. BM was collected from the tibia and femurs of C57BL/6 × 129S4/SvJae F1 CerS2-null mice (expressing only CD45.2^+^) and WT mice and cells (excluding erythrocytes) were counted using a hemocytometer. CerS2-null mice and WT recipients were irradiated with 1,000 cGy and given antibiotics (ciprofloxacin, 1.0% vol/vol) in their drinking water for 2 weeks. The next day, mice were injected i.v. with 3 × 10^6^ BM cells. Chimeras were generated by injecting BM cells from WT mice to CerS2-null mice (WT > KO) and CerS2-null mice to WT mice (KO > WT) along with WT > WT and KO > KO controls. Chimeras were analyzed for the presence of donor WT (CD45.1^+^CD45.2^+^) or CerS2 null (CD45.2^+^) *i*NKT cells in the thymus and liver 8–10 weeks after BM transfer.

### SL Analysis

Sphingolipid levels were analyzed by liquid chromatography-electrospray ionization-tandem mass spectrometry (LC ESI–MS/MS) using an ABI4000 quadrupole-linear ion trap mass spectrometer as described previously (39).

### Statistical Analysis

Data are expressed as means ± SEM. An unpaired student’s *t*-test was used to determine statistically significant differences between groups; *p < 0.05, **p ≤ 0.01, and ***p ≤ 0.001.

## Results

### CerS2-Null Mice Are Susceptible to LCMV-WE Infection

**Figure 1:**
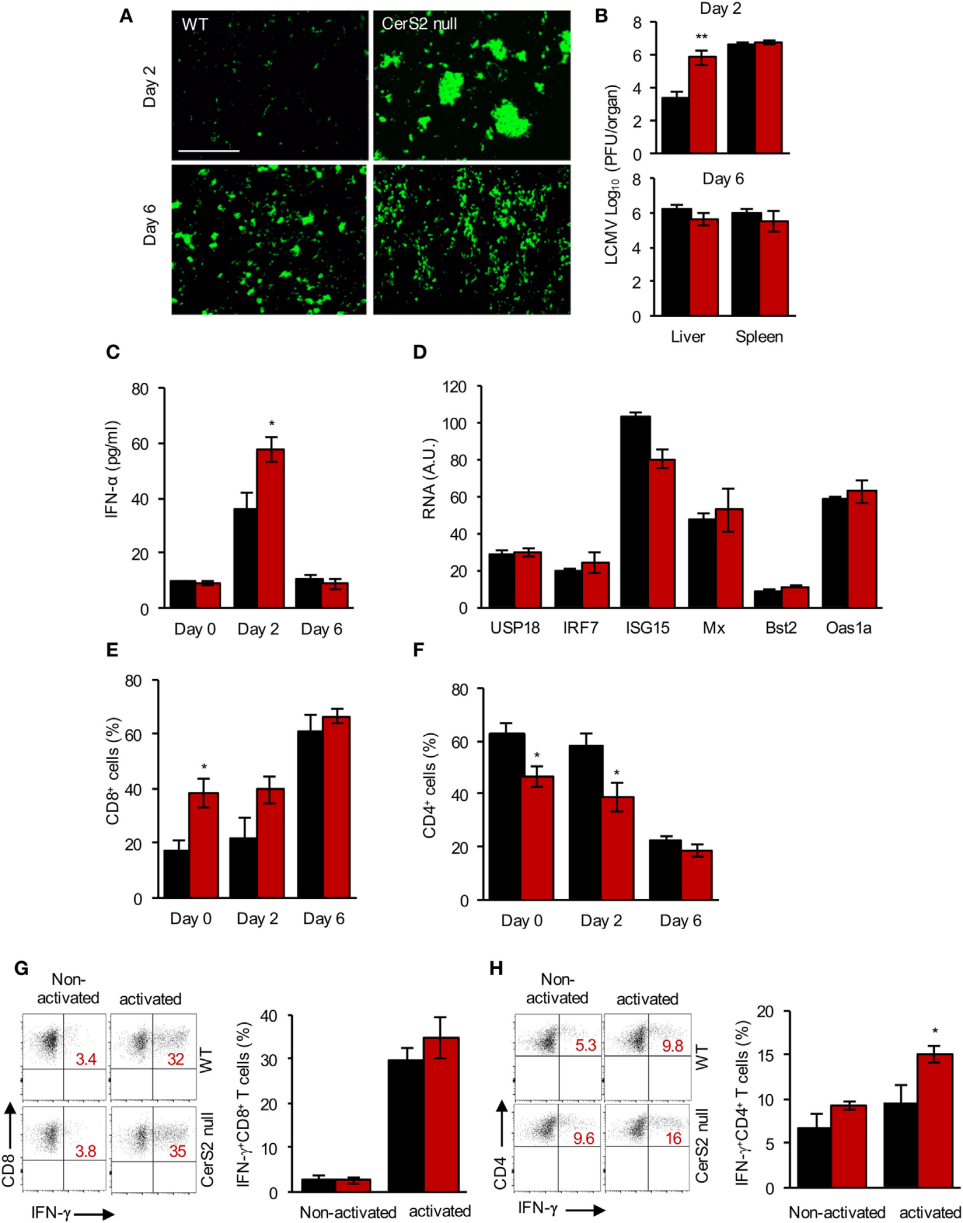
Increased susceptibility of ceramide synthase 2 (CerS2)-null mice to LCMV infection. **(A)** Representative images of wild-type (WT) and CerS2-null liver stained with an anti-LCMV antibody after infection with 2 × 10^6^ plaque-forming units LCMV-WE for 2 and 6 days. Scale bar, 20 µm. **(B)** Viral titers 2 and 6 days post-infection (DPI). The experiment was repeated two times with similar results. **(C)** IFNα levels were measured in a liver homogenate (5 mg protein/ml homogenate) in the liver 2 and 6 DPI. **(D)** qPCR of downstream target genes of IFNR1 signaling 2 DPI in the liver of WT and CerS2-null mice. **(E)** Total CD8^+^ and **(F)** CD4^+^ (percent of total CD3^+^) T cells in the liver of WT and CerS2-null mice 2 and 6 DPI. **(G)** Representative flow cytometry plots and quantitation of intracellular staining of non-activated (without peptide) and activated (LCMV-specific peptide GP33) IFNγ-producing CD8^+^ (percent of total CD8^+^ cells) and **(H)** non-activated and activated (LCMV-specific peptide GP64) CD4^+^ (percent of total CD4^+^) cells. Liver hematopoietic cells from LCMV-infected mice 6 DPI were isolated and restimulated *in vitro* with LCMV-specific peptides. *n* = 3 per group. Black columns, WT; red columns, CerS2 null.

To determine the role of CerS2 in host defense against pathogens, we infected CerS2-null mice with a hepatotropic strain of LCMV, which activates pathways of both innate and adaptive immunity and causes acute hepatitis in mice (40). Two days post-infection (DPI) with 2 × 10^6^ PFU of LCMV, increased levels of the virus were detected in the liver of CerS2-null mice compared to WT mice as ascertained by immunofluorescence (Figure 1A) and by viral titers (Figure 1B). No differences in infection were observed 6 DPI in the liver, and no changes in infection were seen in the spleen at either time point (Figure 1B). Cultured hepatocytes from WT and CerS2-null mice did not display differences in infection excluding the possibility that altered LCMV internalization or proliferative capacity in hepatocytes is the primary cause for the enhanced susceptibility of CerS2-null mice to hepatic LCMV infection (Figure S1A in Supplementary Material).

IFNα is critical for defense against virus infection (41) but, unexpectedly, levels of IFNα were higher in CerS2-null liver 2 DPI compared to WT mice (Figure 1C) and downstream components of IFNAR1 signaling pathway were unaltered (Figure 1D). Likewise, levels of plasmacytoid dendritic cells (pDCs), the major source of IFNα after viral infection (42), were increased in CerS2 null liver (Figure S1B in Supplementary Material). Levels of monocytes were unaltered (Figure S1C in Supplementary Material), whereas neutrophils (Figure S1D in Supplementary Material) and CD8^+^ T cells (Figure 1E) were increased in CerS2-null mouse liver prior to infection, probably due to increased hepatocyte turnover (17). The percent of CD4^+^ T cells was lower in CerS2-null mouse liver before and after viral infection (Figure 1F). However, LCMV-specific CD8^+^ T cells were unaltered (Figure 1G), while LCMV-specific CD4^+^ T cells (Figure 1H) were increased in CerS2 null mouse liver 6 DPI, indicating that impairment of conventional T cell responses cannot explain the enhanced susceptibility. In conclusion, the susceptibility of CerS2 null liver to LCMV infection after 2 days cannot be accounted for by increased viral internalization into hepatocytes, to a type I IFN response or to virus-specific T cell responses.

### Lack of *i*NKT Cells Renders CerS2-Null Mice Susceptible to LCMV Infection

**Figure 2:**
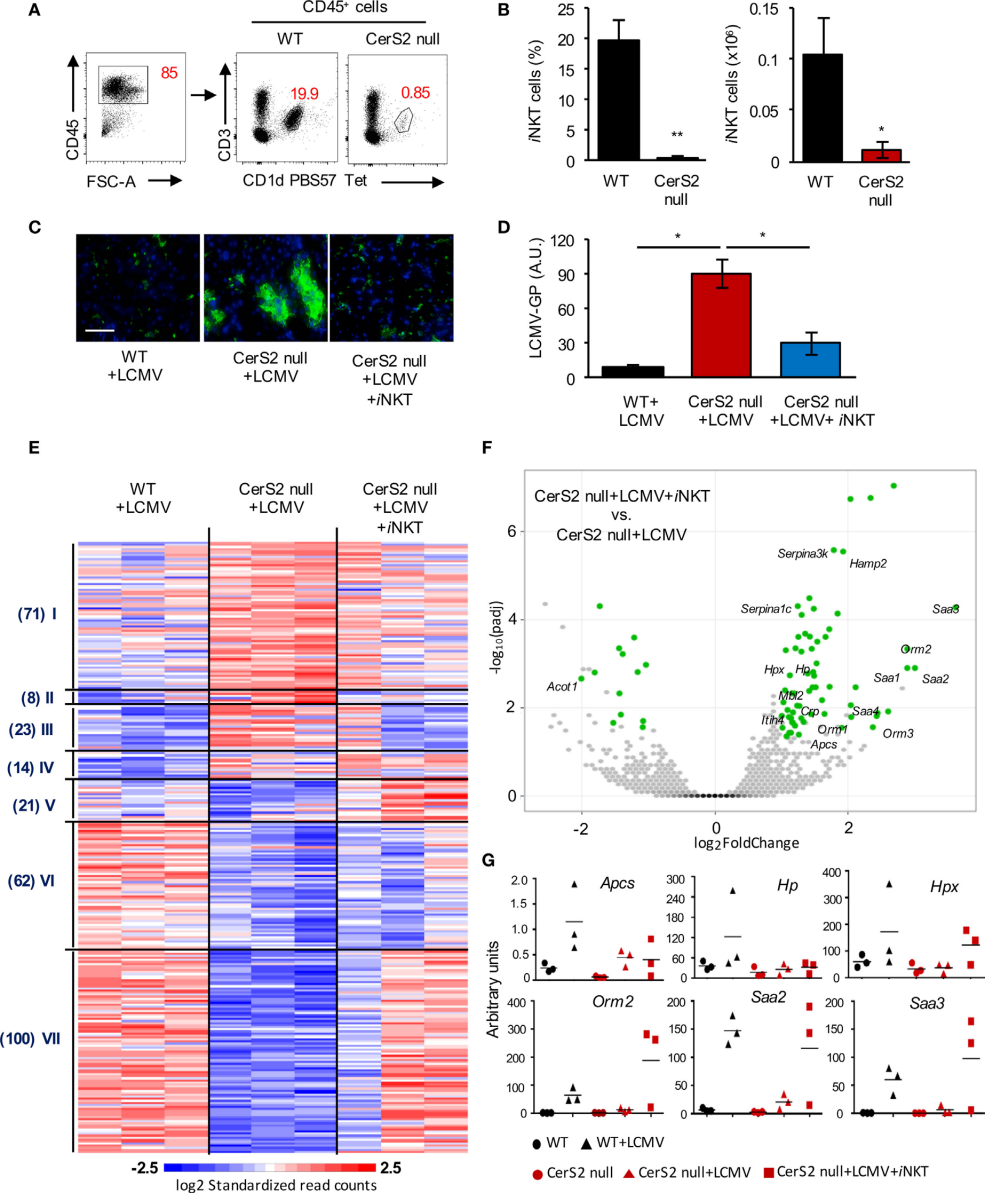
Reduced invariant NKT (*i*NKT) cells in the liver of ceramide synthase 2 (CerS2)-null mice account for their susceptibility to LCMV infection. **(A)** Representative flow cytometry plots showing the percent of CD3^+^CD1d-PBS57 tetramer^+^ *i*NKT cells (indicated by numbers in red) out of total hematopoietic (CD45^+^) cells. **(B)** Percent and absolute numbers of *i*NKT cells (*n* = 4). **(C)** Representative images of viral staining in liver of wild-type (WT), CerS2 null, and CerS2-null mice after adoptive transfer of isolated WT *i*NKT cells 2 days post-infection. Scale bar, 20 µm. **(D)** qPCR of LCMV-glycoprotein (GP). The experiment was repeated three times with similar results. Black columns, WT; red columns, CerS2 null. **(E)** Heatmap of mRNA profiles of liver from LCMV-infected WT, CerS2-null mice, and CerS2-null mice after WT *i*NKT cell transfer (*n* = 3). Differentially expressed genes were clustered using Pearson’s dissimilarity, and the number of partition clusters was set to seven. **(F)** Volcano plot displaying statistical significance (−log10 adjusted *p* value) against the log2 ratio between LCMV-infected CerS2-null mice and LCMV-infected CerS2-null mice after *i*NKT cell transfer, based on RNA-seq data. Significantly changed genes (log2 fold change, adjusted *p* value< 0.05, *n* = 3 for each group) are represented by green symbols. Genes are labeled that were significant and common between LCMV-infected CerS2-null mice/LCMV-infected CerS2-null mice after *i*NKT cells transfer and WT mice before and after LCMV infection but not in CerS2 null before and after LCMV infection. **(G)** qRT-PCR analysis of various differentially expressed genes from cluster VII.

Since the number of CD4^+^ T cells was decreased in CerS2 null mouse liver (Figure 1F), we examined levels of *i*NKT cells, which form a major fraction of hepatic CD4^+^ T cells and are involved in protection against pathogens, including viral infection (43–45). Flow cytometry revealed a striking decrease in the percent and number of *i*NKT cells in CerS2 null liver (Figures 2A,B). To determine if the reduction in the number of *i*NKT cells is responsible for viral infection, mice were engrafted with WT *i*NKT cells 1 day prior to LCMV infection. Adoptive transfer of an enriched *i*NKT fraction abolished the enhanced susceptibility of CerS2-null mice to LCMV infection (Figures 2C,D), while the *i*NKT-depleted fraction (containing high levels of conventional T cells and low levels of *i*NKT cells) did not (Figure S3 in Supplementary Material).

To further delineate the role of *i*NKT cells in hepatic LCMV infection, we performed 3′UTR digital gene expression RNA-seq (Data Sheet S1 in Supplementary Material) and compared genes that were differentially expressed between LCMV-infected CerS2 null mouse liver before and after transfer of *i*NKT cells, with differentially expressed genes in WT mice before and after infection (Figure 2E). Differentially expressed genes that were common in both comparisons, but were not common between CerS2-null mice with and without infection, were considered to be a downstream effect of *i*NKT cells (Figure 2F). Transfer of *i*NKT cell into CerS2-null mice altered the expression of some genes similar to those of WT infected liver (Figure 2E, mainly in cluster VII). Since *i*NKT cells contribute a small fraction of the total RNA (as only 10^6^ *i*NKT cells were transferred), most of the genes whose expression was altered are hepatocyte derived (Figure 2F). Increased expression of several acute-phase reactants, such as amyloid A proteins (*Saa1, Saa2, Saa3*), amyloid P component serum (*Apcs*), haptoglobin (*Hp*), hemopexin (*Hpx*) and orosomucoid 2 (*Orm2*), was observed only in CerS2-null mice after transfer of *i*NKT cells and infection with LCMV (Figure 2F). Changes in gene expression were validated for selected acute-phase genes by qRT-PCR, and compared to uninfected mice (Figure 2G). While expression of *Hpx, Orm2, Saa2*, and *Saa3* were increased upon transfer of WT *i*NKT cells into LCMV-infected CerS2-null mice, *Apcs*, and *Hp* were not increased, indicating that these genes are not influenced directly by *i*NKT cells (Figure 2F).

### The Development of CerS2 Null *i*NKT Cells Is Arrested in the Thymus

**Figure 3:**
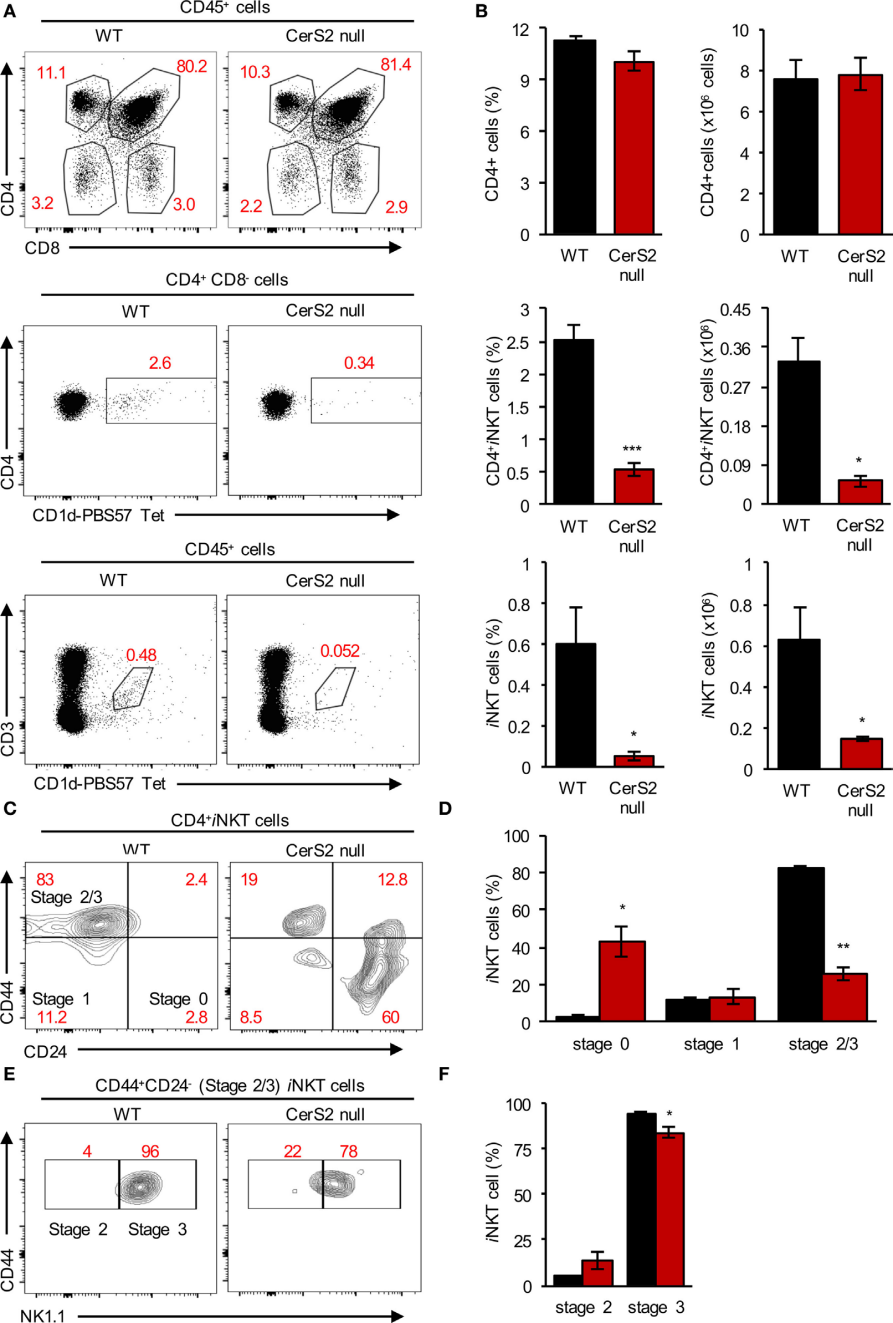
Reduced levels of invariant NKT (*i*NKT) cells in the thymus of ceramide synthase 2 (CerS2)-null mice is due to their impaired maturation in the thymus. **(A)** Representative flow cytometry plots showing the frequencies of thymocytes (*upper panel*), CD4^+^CD1d-PBS57 tetramer^+^ *i*NKT cells (*middle panel*), and CD3^+^CD1d-PBS57 tetramer^+^ *i*NKT cells (*lower panel*) out of total hematopoietic cells in wild-type (WT) and CerS2-null mice. **(B)** Average percent and absolute number of the indicated cells (*n* = 5). **(C,E)** Representative flow cytometry contour plots showing the percent of different developmental stages of *i*NKT cells in the thymus. **(D,F)** Average percent of *i*NKT cells at different developmental stages in the thymus (n = 3). The experiment was repeated two times with similar results. Percents are indicated in red. Black columns, WT; red columns, CerS2 null.

Lower levels of *i*NKT cells were also detected in the thymus of CerS2-null mice, while levels of conventional T cells were unaltered (Figures 3A,B). *i*NKT cells were arrested in their development at the immature DP stage since CerS2-null mice accumulated immature CD24^+^CD44^−^ *i*NKT cells (stage 0) and had less mature CD24^−^CD44^high^ *i*NKT cells (stage 2/3) (Figures 3C,D). Further analysis revealed that most of the cells which were CD24^−^CD44^+^ were at stage 3 (NK1.1^+^ cells) in both WT and CerS2-null mice (Figures 3E,F; Figure S4 in Supplementary Material). However, in CerS2-null mice, there was a small but non-significant increase in stage 2 *i*NKT cells (Figures 3E,F). The arrest of CerS2 null *i*NKT cell maturation could reflect a cell-intrinsic defect in the survival or proliferation of *i*NKT cells as they mature in CerS2-null mice, or alternatively could be due to extrinsic defects such as reduced surface expression of CD1d or lack of lipid self-antigens allowing thymic positive selection of *i*NKT cells.

**Figure 4:**
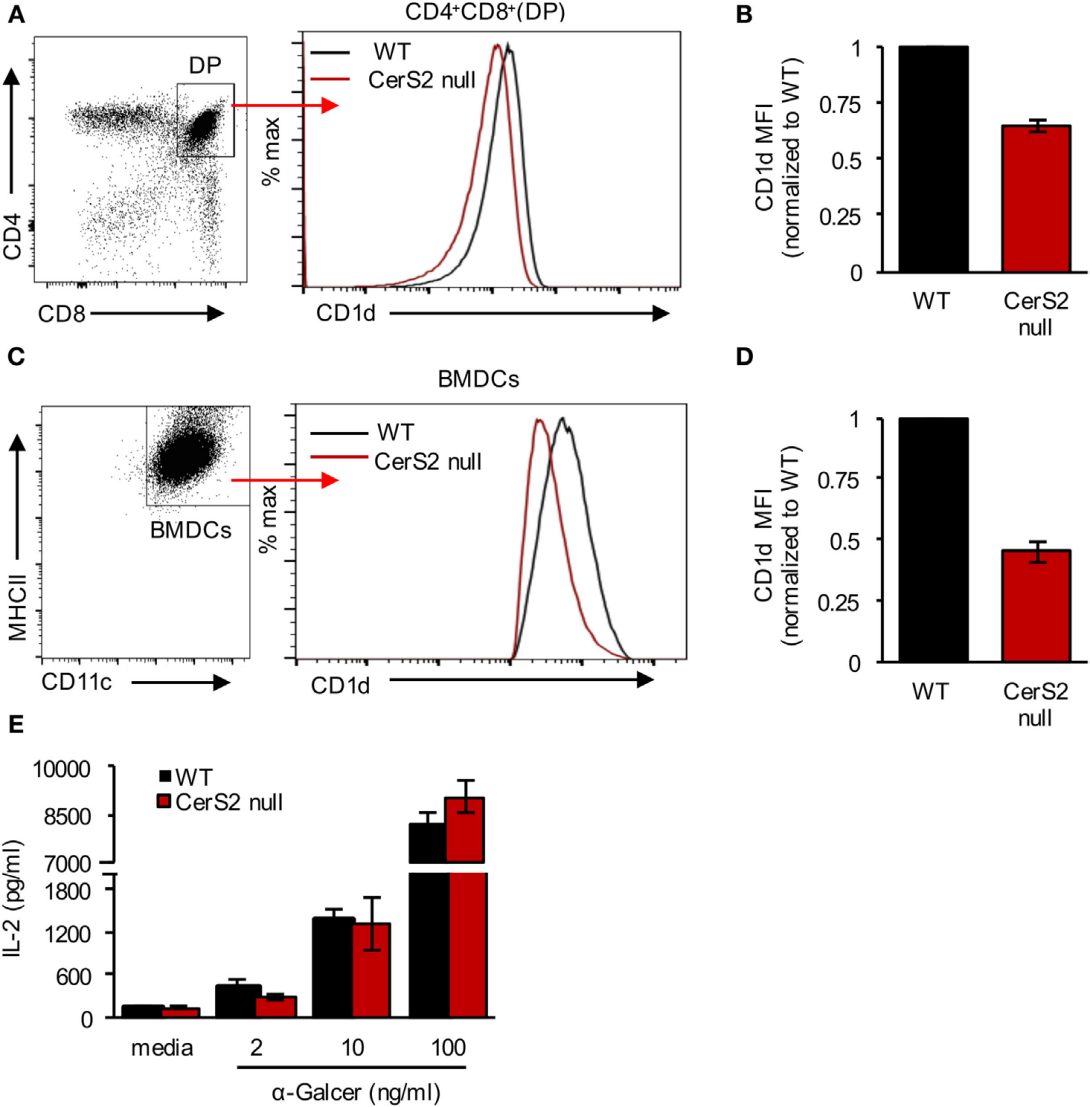
CD1d expression in ceramide synthase 2 (CerS2)-null mice. **(A)** Representative flow cytometry plot of CD4^+^CD8^+^ DP thymocytes (*left panel*) and histogram showing surface expression of CD1d on DP thymocytes in the thymus of wild-type (WT) and CerS2-null mice (*right panel*) **(B)** Quantitation of mean fluorescence intensity (MFI) of CD1d on DP thymocytes in WT and CerS2-null mice (*n* = 4). **(C)** Representative flow cytometry plot of BMDCs (*left panel*) and histogram (*right panel*) showing CD1d surface expression on BMDCs derived from WT and CerS2-null mice. **(D)** Quantitation of MFI of CD1d on BMDCs derived from WT and CerS2-null mice (*n* = 3). The experiment was repeated three times with similar results. **(E)** Activation of DN32.D3 NKT hybridoma cells by α-GalCer incubated with BMDCs. The experiment was performed three times in triplicate with similar results. Black columns, WT; red columns, CerS2 null.

Unlike conventional T cells, *i*NKT cells are selected *via* the presentation of lipid self-antigen(s) by CD1d on DP thymocytes (46, 47). CerS2 null DP thymocytes exhibited a 34 ± 1.5% reduction in CD1d surface expression (Figures 4A,B). Our previous studies demonstrated that surface expression of a number of receptors is reduced in CerS2-null mice (18, 25, 26). To directly test the impact of reduced levels of CD1d on *i*NKT cells, we generated BM culture-derived dendritic cells (BMDCs). In response to α-GalCer, CerS2-null BMDCs, although they displayed a 48 ± 4% reduction in surface expression of CD1d (Figures 4C,D), activated an *i*NKT cell hybridoma cell line (DN32.D3) to a similar extent as WT controls (Figure 4E). Moreover, CD1d^+/−^ mice, which have a 50% reduction in surface CD1d expression on DP thymocytes, contain a normal *i*NKT cell compartment (46). We conclude that the reduced expression of CD1d in CerS2 null DP thymocytes is unlikely to account for the reduced number of *i*NKT cells.

### Reduced Levels of *i*NKT Cells in CerS2-Null Mice Likely Results from Lack of Self-antigens

**Figure 5:**
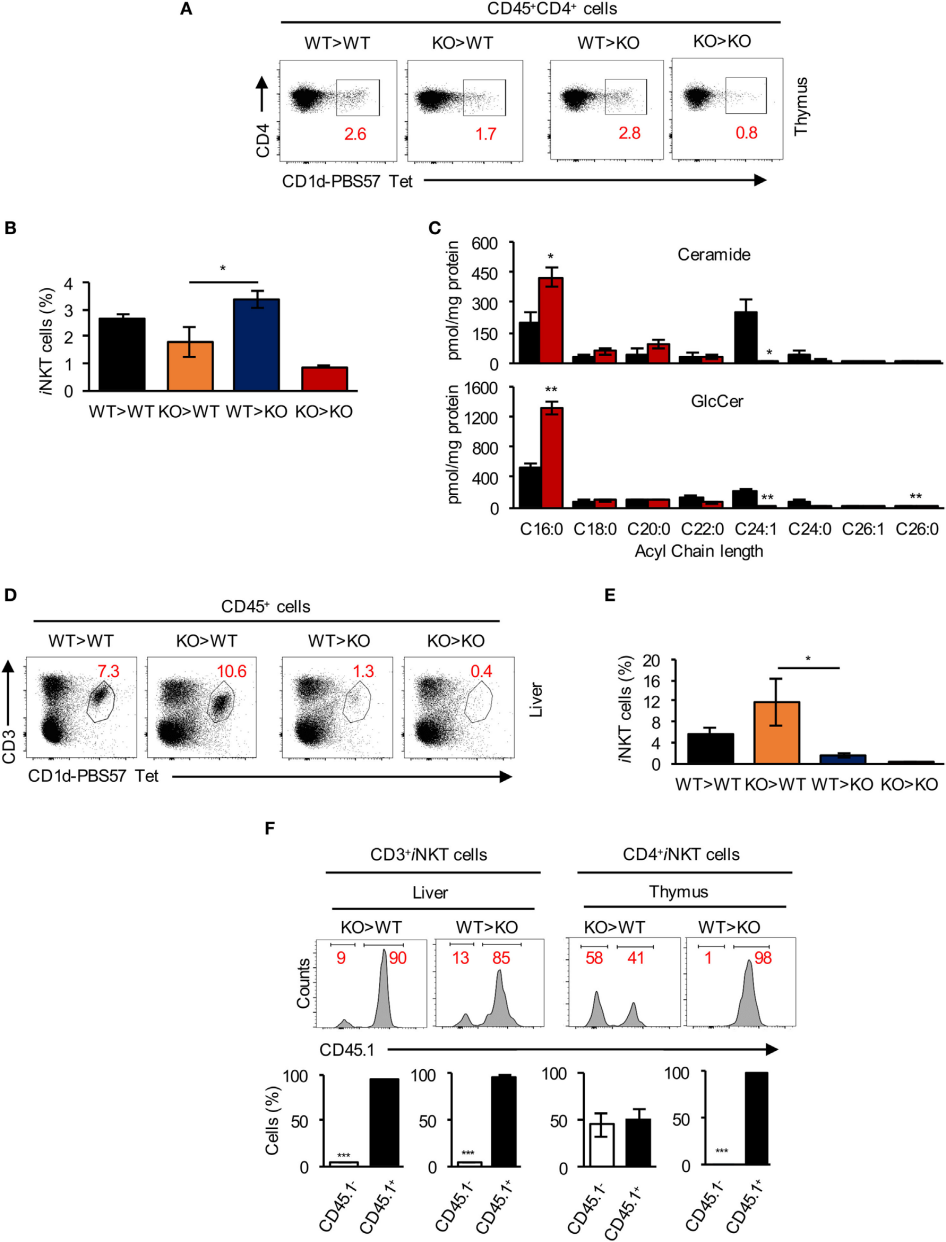
Survival signals from non-hematopoietic cells are required for invariant NKT (*i*NKT) cell survival in the liver. **(A)** Representative flow cytometry plots showing the frequency and **(B)** average frequency of *i*NKT (out of total CD4^+^ hematopoietic cells) in the thymus of bone marrow (BM) chimeras. **(C)** Quantification of the acyl chain lengths of ceramide (*upper panel*) and glucosylceramide (GlcCer) (*lower panel*) in thymocytes of wild-type (WT) (black) and ceramide synthase 2 (CerS2) null (red) mice. **(D)** Representative flow cytometry plots showing the frequency and **(E)** average frequency of CD3^+^ *i*NKT cells (out of CD45^+^ cells) in the liver of BM chimeras. **(F)** Representative flow cytometry histograms of BM chimeras, where WT cells are CD45.1^+^ and CerS2-null cells are CD45.1^−^ [numbers in red indicate the percent of total CD3^+^ *i*NKT cells in liver and CD4^+^ *i*NKT cells in thymus (*upper panel*)]. Quantitation of CD45.1 staining of *i*NKT cells in liver and thymus of CerS2-null mice (*lower panel*). *n* = 2–4 in each group of mice. The experiment was repeated twice with similar results.

We next generated BM chimeras by transfer of WT BM into irradiated CerS2 null hosts (WT > KO) and *vice versa* (KO > WT). WT > KO and WT > WT chimeras had a similar percent of *i*NKT cells in the thymus (Figures 5A,B). In contrast, levels of *i*NKT cells in KO > WT chimeras were reduced and similar to levels found in KO > KO chimeras (Figures 5A,B). These results demonstrate that CerS2 expression by hematopoietic cells is required for thymic *i*NKT cell development.

Consistent with changes in the SL composition in other organs in CerS2-null mice (16, 21, 23), levels of VLC-ceramide and VLC-GlcCer were reduced in CerS2 null thymocytes (Figure 5C). However, GalCer levels were below the limit of detection (0.05 pmol/mg protein) in WT and CerS2-null thymocytes.

While thymic *i*NKT cells in WT > KO chimeras were unaffected (Figures 5A,B), levels of *i*NKT cells in the liver of WT > KO chimeras were reduced to similar levels as found in KO > KO chimeras (Figures 5D,E). Moreover, KO > WT chimeras displayed normal *i*NKT cell levels in the liver, similar to WT > WT chimeras (Figures 5D,E), indicating that radiation-resistant parenchymal cells are essential for *i*NKT cell survival and maintenance in the liver.

Further analysis of BM chimeras using CD45.1^+^ WT donors revealed that in KO > WT chimeras, ~90% of the *i*NKT cells in the liver were WT-derived (Figure 5F). This is likely due to incomplete depletion of host T cells during irradiation (48, 49), which also occurs for conventional T cells but not for B cells (Figure S2A in Supplementary Material), perhaps indicating that CerS2 null T lymphocytes (including *i*NKT cells) have a reduced capacity to compete with residual WT cells (Figure 5F). In the thymus, although very few CD4^+^ *i*NKT cells were recovered in KO > WT chimeras (Figure 5A), ~50% were KO-derived (CD45.1^-^ cells; Figure 5F), probably due to antigen presentation by residual WT DP thymocytes, allowing limited CerS2 null *i*NKT cell maturation. However, these cells were nearly completely absent in the liver of KO > WT chimeras (<10%; Figure 5F). In conclusion, the chimera experiments reveal that CerS2-deficient conventional T and *i*NKT cells have an intrinsic defect when competing with WT cells. However, in CerS2-null mice, where no competition with WT cells occurs, this is unlikely to be the reason for reduced levels of *i*NKT in the thymus, which are arrested at the DP stage when positive selection takes place.

## Discussion

In the current study we provide evidence that generation and maintenance of *i*NKT cells depends on levels of VLC-SLs. The reversibility of the susceptibility to LCMV infection upon adoptive transfer of WT *i*NKT cells into CerS2-null mice strongly supports this contention. These results are in agreement with reports showing that increased levels of *i*NKT cell in the liver are protective against early stages of LCMV infection (50, 51), consistent with a critical role for *i*NKT cells in the early stages of hepatic viral infection (52). The redundant role of *i*NKT cells in splenic viral infection (51, 53) explains why CerS2 null mouse spleen was not more susceptible to LCMV infection.

We demonstrate that *i*NKT cells promote the secretion of Saa1 and other acute-phase proteins in the early stage of viral infection in the liver. Acute-phase proteins are secreted from hepatocytes upon infection to control infection and restore homeostasis (54). Saa1 switches neutrophils from an anti- to a pro-inflammatory state and promotes their interaction with *i*NKT cells (55). *Saa1* and *Saa3* are increased upon HCV infection, while *Acot1*, which hydrolyzes long chain fatty acyl CoAs was downregulated (56), similar to our results upon *i*NKT cell transfer into CerS2 null infected mice (Figure 2F). Together our results suggest that CerS2-null mice lacking *i*NKT cells cannot control viral propagation in part due to lack of specific acute-phase proteins and possibly also due to other pathways such as those involved in lipid metabolism.

When considering the role of GSLs in the generation and activation of *i*NKT cells, three issues need to be addressed, namely, the acyl chain length of the GSL, the type of the glycosyl head group, and the anomeric configuration of the glycoside bond. For the first issue, previous studies suggested a role for the SL acyl chain length in binding to CD1d and in the affinity of this complex for the *i*NKT TCR, with both the long chain base and the acyl chain length determining the stability of lipids bound to CD1d molecules and *i*NKT activation (50, 57, 58). Our study supports the idea that VLC-GSLs are major endogenous ligands necessary for *i*NKT cell maturation in the thymus (59, 60) and maintenance in the liver where *i*NKT cells seek foreign lipids antigens within hepatic sinusoids. In respect to the headgroup of the glycolipid, mice deficient in β-GalCer synthase have normal *i*NKT cell levels and their antigen-presenting cells (APC) can activate an *i*NKT hybridoma cell line, while cell lines deficient in β-GlcCer synthase cannot (61). Moreover, depletion of GlcCer-derived GSLs in DP thymocytes resulted in ~50% reduction of the *i*NKT cell population in the thymus and periphery (62). This supports the notion that C24:1-GlcCer, or more complex GSLs, are the major self-antigens required for *i*NKT cell development in the thymus and that lack of VLC-SLs in CerS2 null antigen-presenting DP thymocytes leads to reduced levels of *i*NKT cells. Concerning the anomeric configuration, previous studies have suggested that an α-linked monoglycosylceramide with a C24:1 acyl chain might be an endogenous ligand of *i*NKT cells (4), although the existence of α-GlcCer in mammals is still somewhat controversial (5, 14) whereas C24:1-β-GlcCer is found at high levels in mouse thymus (63). Our data showing lower levels of VLC-GlcCer, concomitant with lower levels of *i*NKT cells, suggest that VLC-GlcCer, or complex VLC-GSLs, act as the endogenous self-antigens presented by CD1d molecules.

The precise mechanism by which *i*NKT cell maturation is affected in the thymus of CerS2-null mice is currently unknown. However, even though a number of cell surface receptors are mislocalized in CerS2-null mice (18, 25, 26), we can exclude the possibility that changes in cell surface expression of CD1d are responsible for the lower levels of *i*NKT cells in CerS2-null mice. However, we cannot rule out other intrinsic defects in *i*NKT cells. For example, the increased levels of C16-SLs in CerS2 null thymocytes (Figure 5C) might affect lysosomal loading of other self-antigens onto CD1d (64), as occurs in some mouse models of lysosomal storage diseases (59), where massive accumulation of SLs and GSLs occurs (65). However, given the relatively low level of C16-SL accumulation in CerS2 null thymocytes (<2-fold; Figure 5C), we consider this unlikely. Not only are VLC-GSLs required for the maturation of *i*NKT cells in the thymus, our data show that they are also appear to be essential for *i*NKT cell survival and maintenance in the liver. However, previous studies have shown that in the absence of CD1d, *i*NKT cells can still proliferate in the liver although to a lower extent (66–68). Thus, it seems that survival of *i*NKT cells in the liver is a combined effect of antigen presentation by CD1d, as well as other cofactors that may also be deficient in CerS2-null mice. However, reduced expression of known factors required for *i*NKT cell recruitment and survival, such as CXCL16 (69), IL-15 (67), IL-7 (70), and ICOSL (71) was not observed in CerS2 null mouse liver (17). Recently, CerS2 was shown to regulate sphingosine 1-phosphate (S1P) levels and S1P-dependent egress of mature thymocytes from the thymus to the periphery (72), with increased levels of mature thymocytes found in the thymus of CerS2-null mice, which could not exit due to the lack of a S1P gradient. Our study did not show significant changes in levels of mature thymocytes in CerS2-null mice (Figure 3A), which could be due to the different genetic backgrounds of the CerS2-null mice used in the two studies. Irrespective, this is unrelated to the reduced levels of *i*NKT thymocytes in CerS2-null mice, which results from an arrest in maturation arrest at the DP stage (Figures 3C,D).

In conclusion, our data suggest that VLC-SLs (such as C24:1-GSLs) are endogenous self-antigens recognized by developing *i*NKT cells in the thymus and highlight the importance of parenchymal VLC-GSLs, which might also be required for *i*NKT cell survival in the liver.

## Ethics Statement

This study was carried out in accordance with the recommendations of international guidelines. The protocol was approved by the Institutional Animal Care and Use Committee (IACUC).

## Author Contributions

AS and YP-J performed and designed the experiments and wrote the manuscript. NSF performed the RNA seq and EF did the bioinformatics analysis. PS analyzed viral titers. AM and SK carried out ESI–MS/MS analyses of SLs. YJ performed in vitro iNKT cell activation experiments, and FT and CP helped write the manuscript. KL provided the LCMV-WE virus, infected mice, and helped write the manuscript. AHF supervised and funded the studies and wrote the manuscript.

## Conflict of Interest Statement

The authors declare that the research was conducted in the absence of any commercial or financial relationships that could be construed as a potential conflict of interest.

## Supplementary Material

Figure S1 Analysis of hepatocytes and immune cells in ceramide synthase 2 (CerS2)-null mice after LCMV infection. **(A)** Representative images of LCMV-infected hepatocytes (MOI 1:100) from wild-type (WT) and CerS2-null mice 12, 16, 20, 24, and 48 h post-infection. **(B)** Frequencies of CD45^+^CD11b^−^CD11c^+^PDCA1^+^ pDCs, **(C)** CD45^+^CD11b^+^Ly6C^+^ monocytes, **(D)** CD45^+^CD11b^+^ Ly6G^+^ neutrophils in the liver of WT and CerS2-null mice 2 and 6 DPI. *n* = 2–3 in each group. Experiments were repeated at least twice with similar results.

Figure S2 CD45.1 staining in liver and thymus of bone marrow (BM) chimeras. **(A)** Representative flow cytometry histograms (*upper panel*) and average frequencies (*lower panel*) showing CD45.1^−^ and CD45.1^+^ staining (numbers in red are percent of total cells) in liver and **(B)** thymus of BM chimeras. Wild-type (WT) are CD45.1^+^ and ceramide synthase 2 (CerS2) null are CD45.1^−^. *n* = 2–4 in each group. Each experiment was repeated twice with similar results.

Figure S3 The effect of LCMV infection after transfer of wild-type (WT) *i*NKT cell-enriched fractions *versus* transfer of the *i*NKT-depleted fraction. **(A)** Representative flow cytometry plots showing the purity of the bound fraction enriched for *i*NKT cells and **(B)** the unbound fraction rich in conventional T cells. Red numbers represent percent of gated cells. **(C)** Representative images of LCMV staining in liver sections of ceramide synthase 2 (CerS2)-null mice 2 days post-infection after transfer of the bound (*n* = 3) and **(D)** unbound (*n* = 2) cell fractions.

Figure S4 NK1.1 staining on *i*NKT cells from C57BL6 and F1 mice. **(A)** Representative flow cytometry contour plots showing gating strategy for NK1.1 positive and negative *i*NKT cells in C57BL/6, and F1 (C57BL/6 × 129S4/Jae) wild-type (WT) mice. Unstained control staining included all reagents (including SA-APC) used for all the other staining except for bio-anti-NK1.1 **(B)** Intensity of NK1.1 expression in *i*NKT cells in C57 BL/6, and F1 WT mice and WT unstained negative control (*n* = 3).

Data Sheet S1 Raw RNAseq data and analysis of differentially expressed genes in livers isolated from wild-type (WT) and ceramide synthase 2 (CerS2)-null mice with and without LCMV infection, and LCMV-infected CerS2-null mice after transfer of WT *i*NKT cells. The data have been uploaded to Gene Expression Omnibus (GEO), accession number GSE104205.
