# Supplementary figures and images for: Critical Role for Very-Long Chain Sphingolipids in Invariant Natural Killer T Cell Development and Homeostasis
Source: Front Immunol. 2017 Nov 1;8:1386. doi: 10.3389/fimmu.2017.01386 (PMC5672022; doi:10.3389/fimmu.2017.01386)

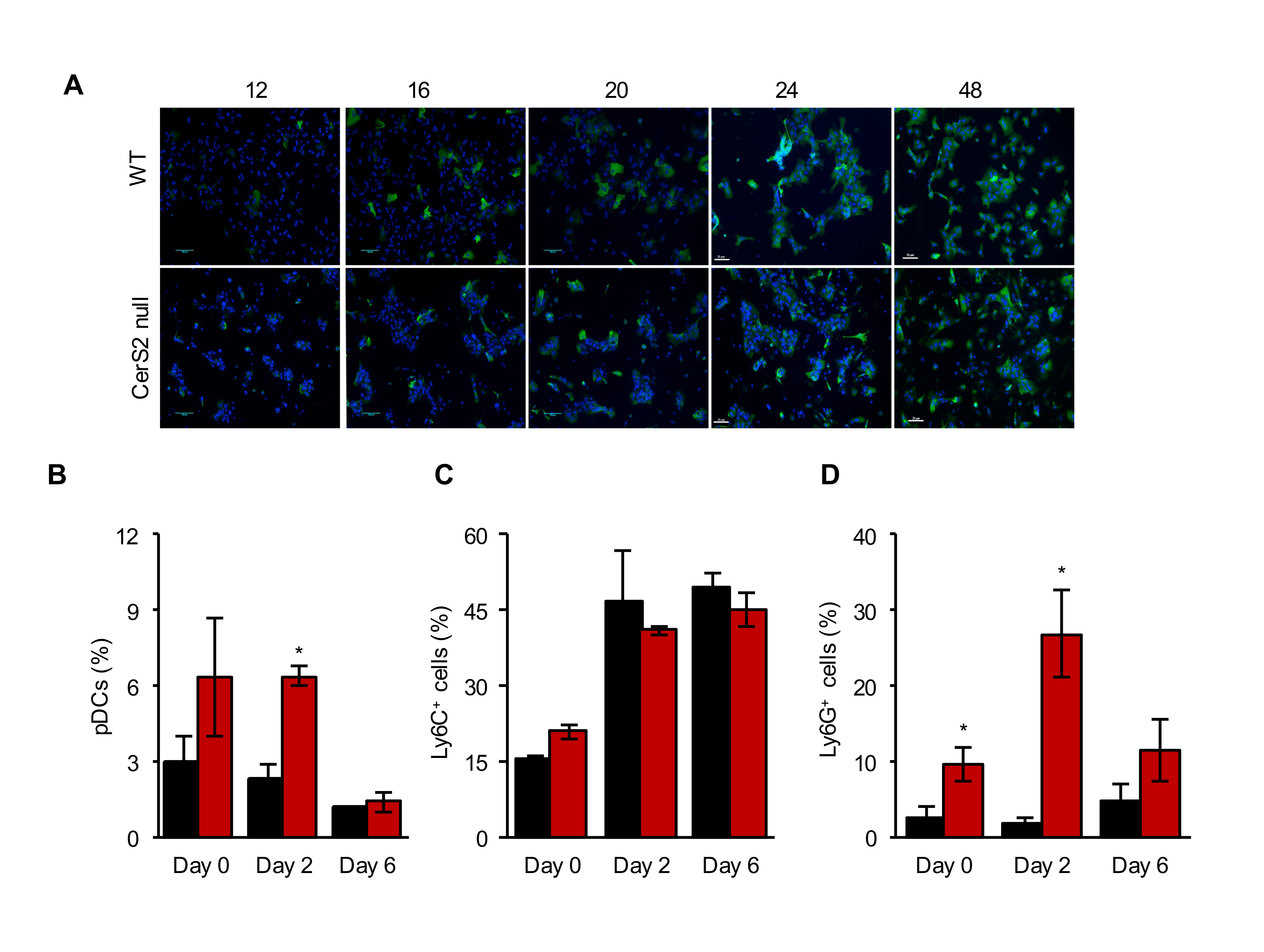

Supplement: Figure S1 — Analysis of hepatocytes and immune cells in ceramide synthase 2 (CerS2)-null mice after LCMV infection. (A) Representative images of LCMV-infected hepatocytes (MOI 1:100) from wild-type (WT) and CerS2-null mice 12, 16, 20, 24, and 48 h post-infection. (B) Frequencies of CD45+CD11b−CD11c+PDCA1+ pDCs, (C) CD45+CD11b+Ly6C+ monocytes, (D) CD45+CD11b+ Ly6G+ neutrophils in the liver of WT and CerS2-null mice 2 and 6 DPI. n = 2–3 in each group. Experiments were repeated at least twice with similar results. [file image_1.tif]

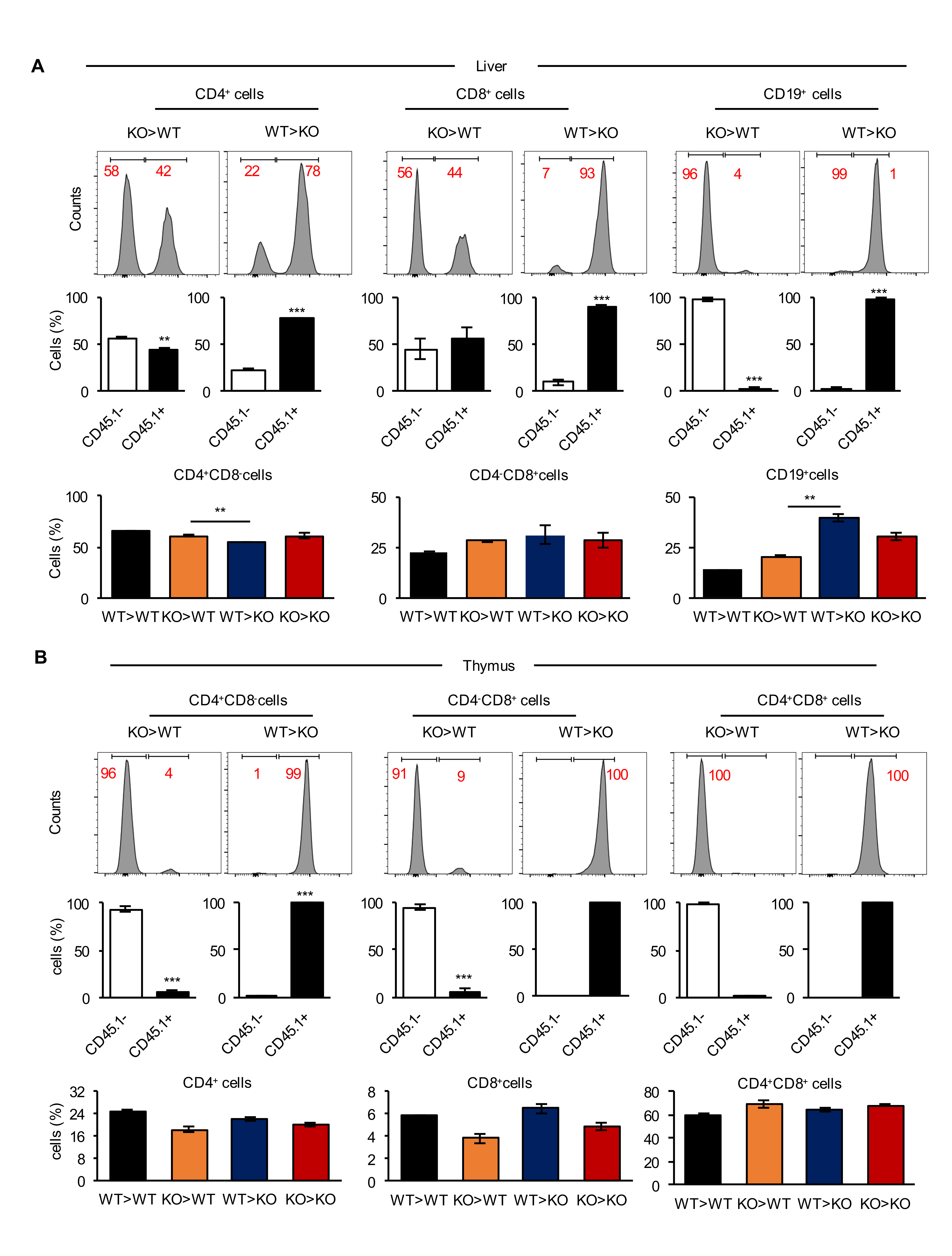

Supplement: Figure S2 — CD45.1 staining in liver and thymus of bone marrow (BM) chimeras. (A) Representative flow cytometry histograms (upper panel) and average frequencies (lower panel) showing CD45.1− and CD45.1+ staining (numbers in red are percent of total cells) in liver and (B) thymus of BM chimeras. Wild-type (WT) are CD45.1+ and ceramide synthase 2 (CerS2) null are CD45.1−. n = 2–4 in each group. Each experiment was repeated twice with similar results. [file image_2.tif]

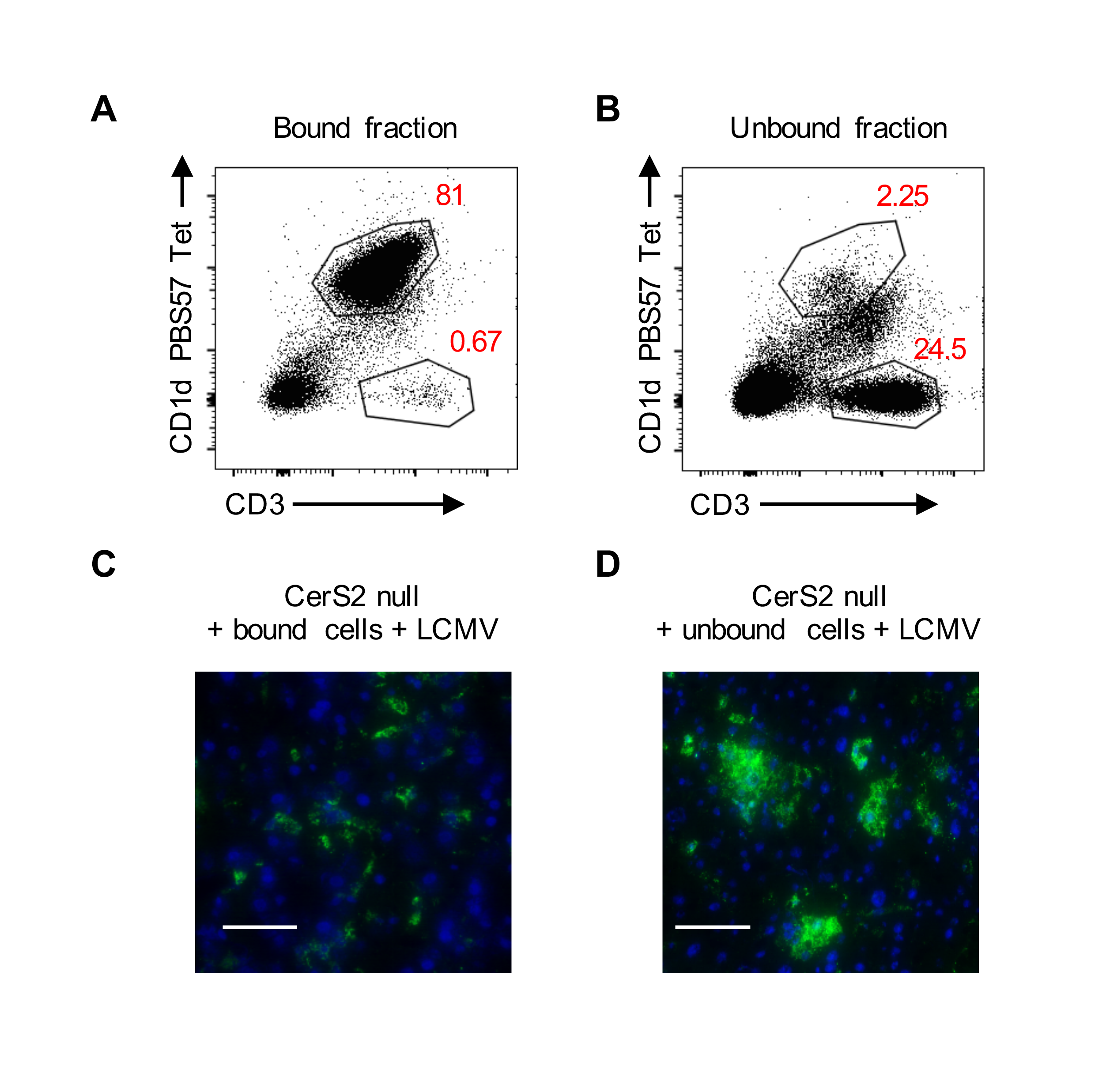

Supplement: Figure S3 — The effect of LCMV infection after transfer of wild-type (WT) iNKT cell-enriched fractions versus transfer of the iNKT-depleted fraction. (A) Representative flow cytometry plots showing the purity of the bound fraction enriched for iNKT cells and (B) the unbound fraction rich in conventional T cells. Red numbers represent percent of gated cells. (C) Representative images of LCMV staining in liver sections of ceramide synthase 2 (CerS2)-null mice 2 days post-infection after transfer of the bound (n = 3) and (D) unbound (n = 2) cell fractions. [file image_3.tif]

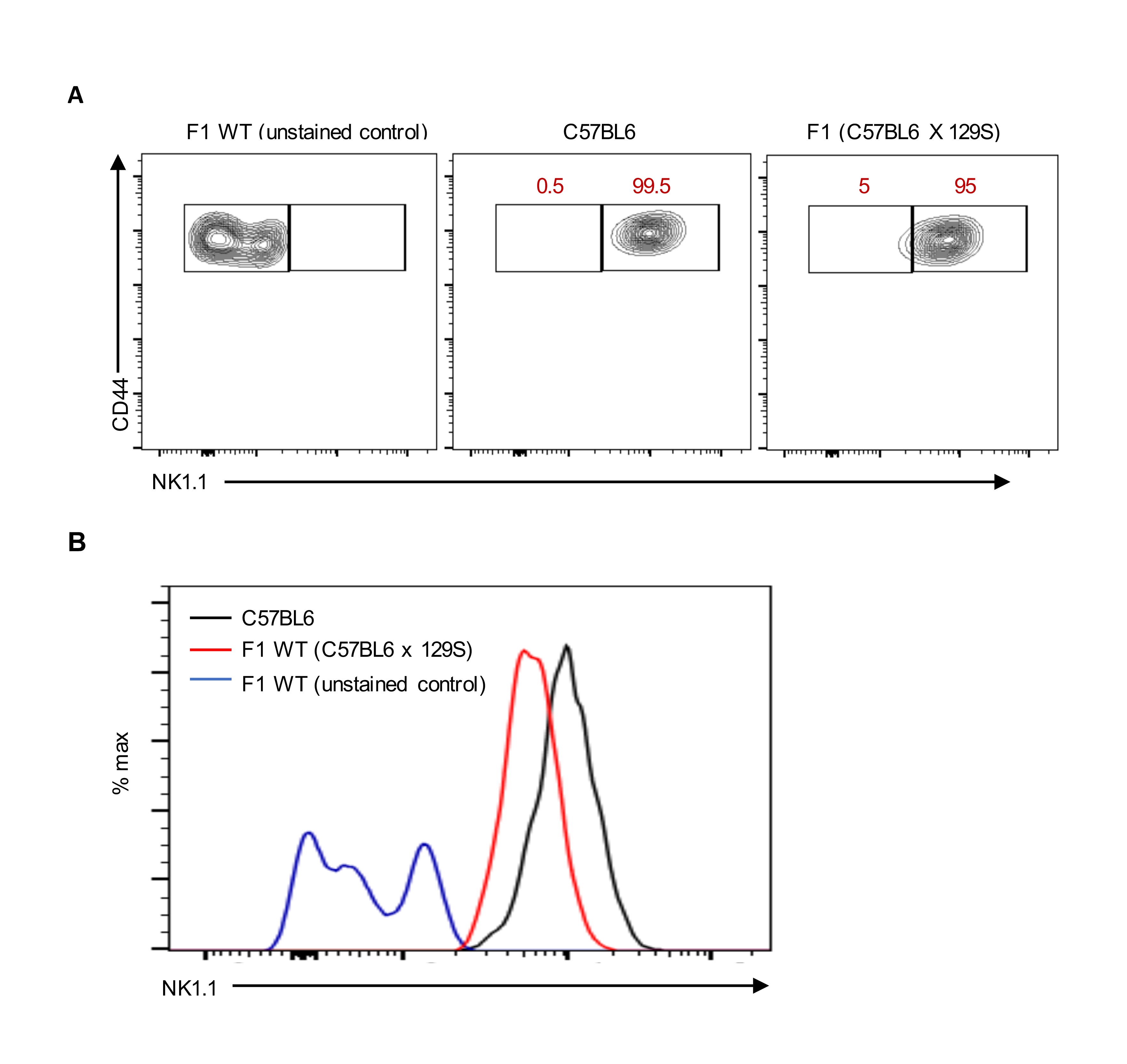

Supplement: Figure S4 — NK1.1 staining on iNKT cells from C57BL6 and F1 mice. (A) Representative flow cytometry contour plots showing gating strategy for NK1.1 positive and negative iNKT cells in C57BL/6, and F1 (C57BL/6 × 129S4/Jae) wild-type (WT) mice. Unstained control staining included all reagents (including SA-APC) used for all the other staining except for bio-anti-NK1.1 (B) Intensity of NK1.1 expression in iNKT cells in C57 BL/6, and F1 WT mice and WT unstained negative control (n = 3). [file image_4.tif]
